# A comprehensive review of oral microenvironment changes and orofacial adverse reactions after COVID‐19 vaccination: The good, the bad, and the ugly

**DOI:** 10.1002/hsr2.1967

**Published:** 2024-03-13

**Authors:** Shaghayegh Najary, Mohammadreza Vatankhah, Gita Khadivi, Seyyede N. Salehi, Mohammad A. K. Tabari, Noosha Samieefar, Mohammad Behnaz

**Affiliations:** ^1^ School of Dentistry Shahid Beheshti University of Medical Sciences Tehran Iran; ^2^ USERN Office Shahid Beheshti University of Medical Sciences Tehran Iran; ^3^ Network of Interdisciplinarity in Neonates and Infants (NINI) Universal Scientific Education and Research Network (USERN) Tehran Iran; ^4^ Center for Craniofacial Molecular Biology, Herman Ostrow School of Dentistry University of Southern California Los Angeles California USA; ^5^ Dentistry Student, Executive Secretary of Research Committee, Board Director of Scientific Society, Dental Faculty Islamic Azad University Tehran Iran; ^6^ Student Research Committee Mazandaran University of Medical Sciences Sari Iran; ^7^ USERN Office Mazandaran University of Medical Sciences Sari Iran; ^8^ Dental Research Center, Research Institute of Dental Sciences, School of Dentistry Shahid Beheshti University of Medical Sciences Tehran Iran

**Keywords:** COVID‐19, Immune system, mucosal disease, SARS‐CoV‐2

## Abstract

**Background and Aims:**

Anti‐severe acute respiratory syndrome coronavirus 2 (SARS‐CoV‐2) vaccines have the potential to alter several biological systems concurrently with remolding the immune system, most of which are related to immunization, while some others are known as adverse effects. This review aims to explore the potential effects of vaccination on the oral microenvironment and classifies them as good, bad, or ugly, with a brief review of facial diseases following coronavirus disease 2019 (COVID‐19) vaccination.

**Methods:**

This study was a comprehensive review conducted through searching related articles in Medline, Scopus, and Google Scholar databases.

**Results:**

On one side, the “Good” impacts of vaccination on the oro‐nasal mucosa are explained as if the mucosal immune responses followed by SARS‐CoV‐2 vaccines are enough to provide immunity. On the other side, the possible “Bad” and “Ugly” effects of the vaccine, which manifest as orofacial adverse events and autoimmune reactivations, respectively, should be noted. Exacerbation of pre‐existing autoimmune conditions such as lichen planus, pemphigus vulgaris, bullous pemphigoid, and Stevens‐Johnson syndrome have been reported.

**Conclusion:**

COVID‐19 vaccines could affect different biological systems alongside stimulating the immune system, and some of these effects are referred to as adverse effects. Nonetheless, these adverse effects are treatable, and healthcare professionals should not prevent patients from taking the first available vaccination.

## 1 BACKGROUND

The coronavirus disease 2019 (COVID‐19) pandemic, given rise by the severe acute respiratory syndrome coronavirus 2 (SARS‐CoV‐2) coronavirus, began in late 2019 in Wuhan, China. It is the most recent infectious disease which has led to a global challenge. Although social distancing and some other transmission prevention approaches have protected many individuals from getting infected,^1^, ^2^, ^3^ safe and effective vaccines and a worldwide immunization program are possibly the best way to bring us back to the prepandemic normal stage, if possible.^4^, ^5^ Worldwide, two RNA‐based vaccines (mRNA‐1273 Spikevax developed by Moderna and BNT162b2 developed by Pfizer‐BioNTech) and one nonreplicating viral vector vaccine (AZD1222 nCoV‐19 from Oxford‐AstraZeneca) are being used as effective tools significantly preventing viral transmission and severe forms of the disease.^6^

The mucosal immune system plays a key role in defence against viral infection. COVID‐19 is mostly transmitted via the respiratory system, and initially infects respiratory mucosal surfaces, inducing the immune system response.^7^ For the vaccination if the same, the immunity induced could be considered as the “**Good**” aspect of immunization. However, whether the fact that injection of SARS‐CoV‐2 vaccines induce mucosal immune responses,^8^, ^9^ and it is enough to provide immunity is still controversial.

Vaccines could affect different biological systems alongside stimulating the immune system, and some of these effects are referred to as adverse effects.^10^ Oral manifestations of vaccine adverse events, such as erosion, ulcer, or mucositis, represent “**Bad**” effects.^11^ The incidence rate of oral adverse reactions induced by SARS‐CoV‐2 vaccines is low, and oral adverse events are considered to be rare. The high number of cases, although could be attributed to the widespread vaccination, should be thoroughly investigated.^12^ These oral adverse effects after immunization with COVID‐19 could be effectively managed with appropriate treatment.^13^ Clinicians need to have a precise knowledge of the true origins of these adverse reactions to manage patients after accurate diagnosis and provide them with proper treatment.

Last but not least, the “**Ugly**” depicts recurrence or new onset of autoimmune disease, followed by immune provocation, induced by COVID‐19 vaccines. Mucosal hypersensitivity and autoimmunity,^14^ recurrence or emergence of pemphigus vulgaris,^15^ bullous pemphigoid,^16^ oral lichen planus,^17^ and other inflammatory autoimmune diseases.^18^, ^19^ were reported days after vaccine administration. Herein, we summarized the oral effects of COVID‐19 vaccination, classified into three groups: the good, the bad, and the ugly. This study is crucial in the current context of the COVID‐19 pandemic, particularly as the world still grapples with the challenges posed by this infection and the implementation of mass vaccination programs.

## 2 METHODS

This study was a comprehensive review conducted through searching keywords in Medline, Scopus, and Google Scholar databases. Studies related to the potential effects of COVID‐19 vaccination on the oral microenvironment and facial diseases following vaccination were included. Only studies written in English were included.

## 3 THE GOOD

### 3.1 The mucosal immune system response to SARS‐CoV‐2 infection

Even though the COVID‐19 pandemic has been ongoing for years, the role of mucosal immunity in SARS‐CoV‐2 infection has attracted little attention. The mucosal immune system is the greatest component of the whole immune system in terms of immune cell recruitment and immunoglobulin secretion.^20^ Furthermore, as SARS‐CoV‐2 infects primarily the upper respiratory tract (URT) at the front line, it is anticipated that mucosal immune responses will be elicited in the nasopharynx, tonsils, adenoids, and even the oral mucosa.^21^, ^22^, ^23^ B cells incorporated in such mucosal inductive site tissues would either be differentiated into IgA‐secreting plasma cells to produce polymeric IgA (pIgA), which then release as secretory IgA (SIgA), or systemic IgG‐producing B cells to secrete circulating IgG.^24^, ^25^ The main structural protein of the SARS‐CoV‐2 virus to enter the target cell, is the S protein; which is made up of S1 and S2 subunits. S1 subunit has a receptor binding domain (RBD) which provides viral attachment to ACE‐2 receptor of the human cell and an S1 domain.^26^ The RBD and S1 domain have the most immunogenicity and are targeted by humoral or cellular immune system.^27^

### 3.2 The role of IgA

In response to SARS‐CoV‐2 infection, in mucosal inductive site tissues such as nasopharynx‐associated lymphoid tissue (NALT).^28^ or even sublingual sites in the oral cavity,^23^ IgA‐producing mucosal B cells migrate and develop into IgA‐secreting plasma cells.^24^ IgA antibodies against SARS‐CoV‐2 have been reported to be elevated in the nasal secretions and saliva of infected individuals. These findings support the notion that SARS‐CoV‐2 induces mucosal IgA antibody response. Therefore, plasma IgA is suggested to be used as a diagnostic tool for the detection of infection.^29^, ^30^ IgA consists of three molecular forms (secretory, monomeric, and polymeric) with two subclasses (IgA1 and IgA2).^31^ Surprisingly, salivary IgA is weakly matched with serum antibody levels which is partly attributable to the cross‐reactive IgA produced by exposure to viruses other than SARS‐CoV‐2.^32^ Levels of IgA may also represent the degree of disease severity, as IgA production has been reported to be higher in severe patients compared to moderate or asymptomatic individuals.^33^

IgA, secreted by local plasma cells against RBD on the virus's spike protein, potentially neutralizes SARS‐CoV‐2 before it binds to epithelial cells.^34^ SIgA, the most abundant immunoglobulin secreted by mucosal surfaces, has a dimeric structure and comprises variable proportions of IgA1 and IgA2 subclasses. While circulating IgA is primarily monomeric and dominated by IgA1 subclass.^35^ The mucosal immune system creates an SIgA‐dominated environment against viruses in the nasopharynx and the upper respiratory tract and bronchi, which is noninflammatory. However, the terminal airways and alveoli are mostly dominated by circulation‐derived IgG.^7^ Since circulating monomeric IgA cannot be readily conveyed to mucosal secretions, there will likely be separate systemic and mucosal responses against this infection.^36^

### 3.3 Plasma and oral fluids antibody levels following vaccine administration

Plasma IgG and IgA levels have been observed to be elevated for up to 2 months after administration of the first dose of BNT162b2 vaccine, and after two doses of mRNA vaccines (ChAdOx1 nCoV‐19 or BNT162b2 COVID‐19).^37^ Additionally, increased levels of circulating IgM and IgG against S and RBD of SARS‐CoV‐2 were measurable for up to 8 weeks after the booster dose.^38^ Nevertheless, it is commonly assumed that intramuscular or subcutaneous vaccinations do not efficiently generate mucosal immunity compared to inhalable vaccines.^39^ Chan et al.^38^ implied that BNT162b2 (but not Sinovac) increases levels of IgA and IgG antibodies with neutralizing activity in the nasal mucosa alongside the humoral immune system, and hypothesized that this increase reduces the asymptomatic transmission risk of virus.

Saliva has been demonstrated to be a reliable biofluid for detecting the presence of SARS‐CoV‐2 mRNA.^40^, ^41^ and also the salivary glands have been proposed to be as a viral reservoir.^42^ Despite virus replication in the oral cavity, few studies have evaluated anti‐SARS‐CoV‐2 antibodies produced by the oral mucosa or salivary glands. According to a study, mRNA vaccination produces measurable amounts of salivary S1‐RBD IgA and IgG, but the ability to neutralize viral infection remained unclear.^43^ Another research demonstrated that plasma and saliva IgG antibodies against SARS‐CoV‐2 are retained for at least 3 months in the majority of COVID‐19 patients. Based on this correlation between serum and saliva IgG, levels of salivary IgG antibody could be used as an indicator of systemic SARS‐CoV‐2 immunity maintenance.^29^ Additionally, IgA and IgM levels in response to spike and RBD antigens decrease significantly after 3 months, suggesting the fact that IgA levels remain elevated for a short period of time, which neither have diagnostic nor protective value in the long term.^29^

Gingival crevicular fluid (GCF) is a highly concentrated serum effusion that drips into the gingival sulcus and includes critical substances and cells related to the immune system such as antibodies.^44^ Shedding of viruses like the human cytomegalovirus and the herpes simplex virus have been reported in the GCF.^45^ The SARS‐CoV‐2 RNA has been detected in both GCF and saliva with a sensitivity of 63.64% and 64.52% in comparison with nasopharyngeal swab sampling, respectively.^46^ Recently, it has been found that the rate of antibodies in GCF and plasma of COVID‐19‐positive patients is almost the same. This supports the notion that GCF could be used as a noninvasive method for monitoring the immune system status.^47^ Antibody levels against S1‐RBD antigen following the second dose of the mRNA BNT162b2 vaccine were significantly higher in GCF than saliva at every stage, and maximum levels were reached 3 weeks following vaccination.^48^

### 3.4 Mucosal vaccine delivery

It has been demonstrated that vaccines administered by injection are unable to effectively prevent viral infection at mucosal sites, such as the nasopharynx, and do not induce protective oral or nasal mucosal immunity via blocking virus transmission.^9^ Various mucosal noninvasive vaccines have developed (Table 1) to address these issues by stimulating long‐lasting protective immune responses in the mucosal sites where SARS‐COV‐2 infection first enters.^49^ The majority of the COVID‐19 mucosal vaccines under study are intended for intranasal administration. There are now a number of intranasal vaccines that are being tested both in preclinical and clinical settings.^50^ The SARS‐COV‐2 synthetic live attenuated vaccine, COVI‐VAC, is administered intranasal. In contrast to other vaccines, COVI‐VAC protects against all viral antigens, not only spike protein which is likely to be protective against a variety of SARS‐CoV‐2 strains.^51^ AdCOVID is an intranasal adenovirus type 5‐vectored vaccine that encodes the SARS‐CoV‐2 spike protein's RBD. A single intranasal vaccination with AdCOVID resulted in a significant and targeted immune response against RBD through the secretion of mucosal IgA in the respiratory tract, as well as systemic immunity, which lasted for over 6 months.^52^ Zhu et al.^53^ developed a bacteriophage T4‐based mucosal vaccine. It was demonstrated that intranasal administration of two doses of this vaccine, 21 days apart, provided both mucosal and systemic immunity.^53^ Additionally, oral vaccines have proven to successfully induce and activate the mucosal immune system for infectious diseases other than COVID‐19.^54^

**Table 1:** A summary of intranasal and oral vaccines.

Vaccine name	Developer	Delivery route	Vaccine type	Clinical phase
COVI‐VAC^51^, ^55^	Codagenix/Serum Institute of India	Intranasal	Live attenuated	Phase 3
AdCOVID^52^, ^56^	Altimmune, Inc.	Intranasal	Viral vector expressing the RBD spike protein	Phase 1 (Discontinued)
T4‐CoV‐2 nanovaccine^53^	USA	Intranasal	Bacteriophage harboring engineered Spike trimers on capsid exterior and nucleocapsid protein	Preclinical stage
DelNS1‐2019‐nCoV‐RBD‐OPT1^57^	University of Hong Kong, Xiamen University, and Beijing Wantai Biological Pharmacy	Intranasal	Viral vector (Replicating)	Phase 3
CIGB‐669 (RBD+AgnHB)^58^	Center for Genetic Engineering and Biotechnology (CIGB)	Intranasal	Protein subunit	Phase 1/2
Razi Cov Pars^59^	Razi Vaccine and Serum Research Institute	Intranasal	Protein subunit	Phase 3
BBV154, Adenoviral vector COVID‐19 vaccine^60^	Bharat Biotech International Limited	Intranasal	Viral vector (Non‐replicating)	Phase 3
Avacc 10^58^	Intravacc B.V.	Intranasal	Protein subunit	Phase 1
RCVi^58^	Wuhan BravoVax	Intranasal	Viral vector (Non‐replicating)	Phase 1
VXA‐CoV2‐1^61^	Vaxart	Oral tablet	Adenoviral‐vector expressing spike ‘S’ and nucleocapsid ‘N’ proteins	Phase 2
OraPro‐COVID‐19^62^	UK‐based company isoBio	Oral capsule	Non‐replicating viral‐vector that expresses the ‘S’ protein	Preclinical stage
bacTRL‐Spike^TM^ ^58^	Symvivo Corporation	Oral	bacTRL‐Spike oral DNA vaccine	Phase 1
CoV2‐OGEN1, protein‐based vaccine^58^	USSF/Vaxform	Oral	Protein subunit	Phase 1

The tablet version of the vaccine would allow those without access to a vaccination site in underdeveloped countries to be vaccinated without a healthcare provider. Also, it reduces the risk of injectable vaccine's adverse effects, including malaise, discomfort, and inflammation.^63^ As an example, Vaxart is an enteric‐coated tablet vaccine that induces more antiviral SARS‐CoV‐2‐specific T cells, notably IFNg‐producing CD8s, than conventional COVID‐19 mRNA vaccines.^61^

**Figure 1:**
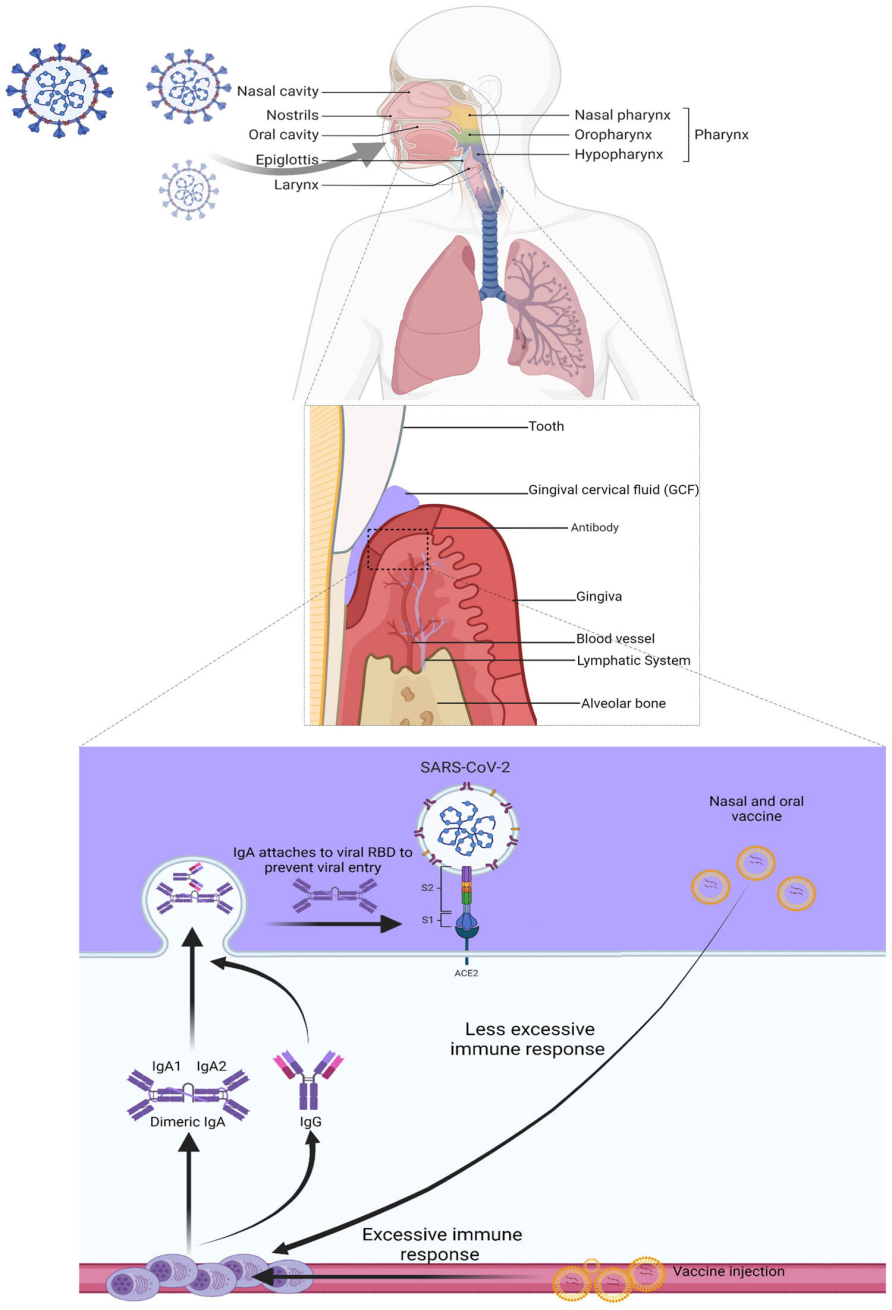
The immune mechanisms involved in mucosal immunity following coronavirus disease 2019 vaccination (comparing oral and nasal with injectable vaccines).

Figure 1. provides a summary of immune mechanisms and a comparison of mucosal vaccine delivery versus injectable vaccines.

## 4 THE BAD

The post‐COVID‐19 vaccination symptoms resemble the post‐COVID‐19 infection condition in many ways. Fatigue has been the most prevalent complaint. More serious complications, including stroke, renal failure, myocarditis, and lung fibrosis have also been reported.^64^ Symptoms more related to the oral cavity have also been recorded, such as oral lesions, mucositis, petechiae, oral mucosal paresthesia or hypoesthesia, candida infection, and necrotizing gingivitis.^12^, ^65^ The exact underlying mechanisms behind such oral manifestations have yet remained to be thoroughly explained. However, some studies suggest the potential of direct SARS‐CoV‐2 invasion of oral keratinocytes, fibroblasts, tongue, and salivary gland epithelial cells as the etiopathogenic mechanism underlying SARS‐CoV‐2 oral adverse effects.^66^ Furthermore, there is growing evidence that anti‐SARS‐CoV‐2 vaccines are related to orofacial side effects.^67^ It is noteworthy to mention that there are discrepancies in the descriptions of vaccine‐related orofacial adverse events between the countries. There is a possibility that oral adverse reactions have been probably underreported by healthcare workers.^68^ However, the overall incidence rate of oral side effects following the anti‐SARS‐CoV‐2 vaccines administration has been reported in a study conducted by Mazur et al.^69^ to be rare with an overall incidence rate of 3.1% and 5.4% after the first and second doses of BNT162b2 vaccine, respectively.

### 4.1 Oral lesions

The majority of the vaccine‐related oral lesions are commonly associated with or secondary to dermatological signs, thus, known as mucocutaneous adverse drug reactions.^69^ Erosion and ulcers have been the most common primary oral lesions observed in adult cases who received the anti‐SARS‐CoV‐2 vaccine, similar to SARS‐CoV‐2‐positive patients. Nevertheless, erythema multiforme and erosive‐ulcerative lesions are mostly attributed to vaccine ADRs, despite aphthous‐like and herpetiform lesions that are considered to be post‐SARS‐CoV‐2 infection complications.^70^

A higher occurrence of oral lesions after receiving anti‐SARS‐CoV‐2 vaccines has been reported in females (68.8%) in comparison to males (31.2%). This is similar to the cutaneous reactions described after the BNT162b2 and mRNA‐1273 vaccinations, which were nine times higher in female subjects.^70^, ^71^ Even after receiving other antiviral vaccines such as anti‐influenza, anti‐Yellow Fever, and anti‐rubella, the incidence of ADRs in females has been found to be higher than in males.^72^

In terms of vaccine type, the BNT162b2 vaccine has had a greater prevalence of cases reported with oral lesions, mostly in the form of erosions and ulcers.^70^ Yet this can be explained by the higher number of BNT162b2 doses delivered in Europe compared with other vaccines.^73^ Oral aphthous‐like lesions have occurred after receiving the first (1.6% of cases) or the second dose (2.7% of cases).^69^ The mRNA‐1273 vaccine is mostly associated with oral lesions in the form of erosions and ulcers similar to BNT162b2, but DNA‐based viral vector vaccines such as ChAdOx1 and Ad26.COV2.S (Janssen) are commonly attributed to oral lesions in the forms of maculae and white plaques, respectively.^70^ Few studies reported the adverse effects of BBIBP‐CorV (Sinopharm) vaccine, which does not necessarily mean that this vaccine causes fewer complications.

### 4.2 Oral mucositis

Oral mucositis can be either be triggered by a direct viral infection of the epithelial cells or induction of immune responses, followed by virus infection or vaccine administration.^74^, ^75^ SARS‐CoV‐2 vaccination provokes a specific adaptive immune response which may underlie the mucosal hypersensitivity, as it has been shown that AZD1222 vaccine induces a T‐cell‐specific response.^75^, ^76^ Furthermore, immune cross‐reactivity may result from similarities between some vaccine components (adjuvants) and human proteins, such as antigens expressed on keratinocytes.^77^ Additionally, it is probable that direct infection of cells expressing angiotensin I converting enzyme 2 receptor (such as epithelial cells of the tongue and salivary glands) by the attenuated or viral vector and rarely mRNA‐based vaccines have contributed to the oral mucositis and ulcerations.^78^, ^79^

### 4.3 Bell's palsy

Facial nerve palsy has also been described as a side effect of vaccine administration, most commonly after the influenza vaccine.^80^ The mRNA‐based vaccines such as BNT162b2 and mRNA‐1273 have been associated with a considerably greater incidence of Bell's Palsy than the Ad26.COV2.S COVID‐19 vaccine.^81^ However, FDA noted that the incidence of Bell's palsy following COVID‐19 vaccination did not exceed that of the general population and refuted a causal relationship between the vaccine delivery and the occurrence of Bell's palsy.^82^, ^83^

### 4.4 Other adverse reactions

The burning sensation occurred as the most common oral adverse effect following both the first and second doses of BNT162b2 vaccine. Additionally, taste alterations frequently occurred after the second dose (3.4%), compared to the first dose (1.1%).^69^ The mRNA‐1273 vaccine has caused rare orofacial ADRs, such as facial swelling in cases with previous cosmetic filler injections in the face or lips.^70^ Moreover, an unusual case of vaccine‐related painful glossitis and xerostomia were recorded following administration of BNT162b2 vaccine which caused further stomatitis and tongue fissures. The symptoms resolved after 2 weeks of applying topical prednisolone.^84^ The allergic reactions might also be induced by ingredients of the vaccine, such as polysorbate 80 and polyethylene glycol (PEG), which makes skin or intradermal injection testing mandatory before administration.^85^ The allergic reaction could also lead to swollen lips, tongue, and mouth.^86^, ^87^

A summary of orofacial adverse reactions following COVID‐19 vaccines is available in Table 2.

**Table 2:** Common and rare orofacial adverse effects following different types of COVID‐19 vaccines.

COVID‐19 vaccine types		Common orofacial adverse effects	Rare orofacial adverse effects	Most frequent forms of oral lesions
DNA‐based viral vector	AstraZeneca (ChAdOx1 nCoV‐19)	Oral mucositis^75^	Palatal petechial lesions^88^	Maculae^70^
	Janssen (Ad26.COV2.S)	Autoimmunologic reaction^89^	Mucocutaneous adverse reaction^90^	White plaques^70^
Attenuated or inactivated virus	Sinopharm (BBIBP‐CorV)	‐	Autoimmunologic reaction.^91^ erythema multiforme^92^	Erosions and erythematous lesions^91^
mRNA‐based vaccines	Pfizer‐BioNTech (BNT162b2 COVID‐19)	Bell's Palsy^81^ Burning sensation^69^	Glossitis and Xerostomia^84^	Erosions and ulcers^70^
	Moderna (mRNA‐1273)	Bell's Palsy^81^	Facial swelling^70^	Erosions and ulcers^70^

## 5 THE UGLY

Recent studies have reported some cases of emergence or exacerbation of autoimmune diseases with symptoms in the oral region after COVID‐19 vaccination. Oral lesions following anti‐SARS‐CoV‐2 vaccinations can be classified as primary oral lesions or immune‐related exacerbation of autoimmune disease, of which the former has been discussed as the bad aspect and the latter will be reviewed below as the ugly consequences (a summary is provided in Table 3).

**Table 3:** Summary of recent studies reporting cases of emergence or exacerbation of autoimmune diseases with oral lesions after COVID‐19 vaccination.

First author	Participant(s) receiving vaccines	Vaccine	Dose number	Type of autoimmune disease	Disease status	Time of onset following vaccination	Signs and symptoms	Lesion characteristics	Treatment	Suggested mechanism
Troeltzsch et al.^89^	A 49‐year‐old male	Ad26.COV2.S	‐	Oral lichen planus	New onset	6 days after vaccination	Oral mucosal discomfort, burning sensations, and desquamation	**Buccal mucosa**: Reticular white markings (Wickham striae) **Togue**: plaque‐like lesions	A 4‐week course of topical clobetasol mouth irrigation solution (0.5 mg/mL)	Vaccine‐induced cytokine flare
Caggiano et al.^93^	A 40‐year‐old male	BNT162b2	Second dose	Oral Lichen Planus	New onset	30 days after vaccination	Bilateral lesions in buccal mucosa	**Buccal mucosa**: Bilateral keratotic reticular patches and erythematous and erosive lesions	‐	Cell‐mediated immune reaction
Picone et al.^94^	A 81‐year‐old male	mRNA‐1273	First dose	Oral and cutaneous lichen Planus	New onset	7 days after vaccination	One week history of intense pruritic eruption	**Buccal mucosa**: symmetrical and bilateral papular whitish lesions	Topical corticosteroids (clobetasol propionate) and H(1)‐antihistaminic therapy (cetirizine 10 mg/daily) for 10 days	T cell‐mediated immune reaction
Bularca et al.^95^	A 29‐year‐old female	BNT162b2	Second dose	Oral and cutaneous lichen Planus	New onset	7 days after vaccination	Lesions on depigmented areas of the hands (patient had vitiligo on her hands)	**Buccal mucosa**: reticular white marking and white plaques	Topical clobetasol propionate and systemic prednisone. Subsequently, treatment with methotrexate 10 mg a week.	T cell‐mediated immune reaction
Kulkarni et al.^96^	A 65‐year‐old female	‐	‐	Oral Lichen Planus	Flare‐up	Immediately following the administration	Increased soreness and inflammation in her left buccal mucosa	‐	‐	T cell‐mediated immune reaction
Kaomongkolgit et al.^97^	A 28‐year‐old female	BNT162b2	Second dose	Oral Lichen Planus	New onset	7 days after vaccination	Six‐week history of oral mucosal discomfort and burning sensations	**Buccal mucosa**: Bilateral papular and reticular white striae	Topical steroid, fluocinolone acetonide 0.1% in orabase paste for 2 weeks	Upregulation of Th1 response
Aryanian et al.^98^	A 43‐year‐old man	ChAdOx1 nCoV‐19	Second dose	Pemphigus vulgaris and lichen planus pigmentosus	New onset	Within one month after the administration of the second dose	Three‐month history of mouth sores, erosive buccal lesions, and dark spots on the face	**Buccal mucosa**: Erosive mucosal lesions	**PV** 80 mg/day prednisolone and 150 mg/day azathioprine then rituximab (500 mg every week for 4 sessions) **LP** topical corticosteroid (mometasone cream qhs)	Provocation of cytotoxic CD8 + T cells and memory cells
Solimani et al.^99^	A 40‐year‐old female	BNT162b2	First dose and Second dose	Pemphigus Vulgaris	New onset and flare‐up	5 days after receiving the first dose and 3 days after the second dose	Painful, non‐healing erosions of the oral mucosa, the trunk, and the back	**Buccal mucosa**: Bilateral extensive painful erosions	Immunosuppressive treatment with oral prednisone (1 mg per kg body weight, eventually tapered) and azathioprine (100 mg/day)	Boosted T/B cell response
Ong et al.^100^	A 46‐year‐old woman	mRNA‐1273	First dose	Pemphigus vulgaris	Flare‐up	7 days after vaccination	Symptoms of acute pemphigus flare‐up	**Buccal mucosa**: Bilateral erosions	Prednisone taper at 1 mg/kg/day then continued with rituximab 1 g on day 1 and day 15. The alternative second dose of Johnson & Johnson vaccine was administered.	Genetics and cross‐reactivity of vaccine antigens with pemphigus‐related/associated antigens
Zhang et al.^101^	An 81‐year‐old man	Inactivated COVID‐19 vaccine	Third dose	Bullous pemphigoid	New onset	15 days after vaccination	Painful erosion of the oral mucosa	Palatal and buccal mucosa: erosive ulcers	Intravenous prednisolone (60 mg/day) and gamma globulin (20 g/day) therapy	Immune predisposition or subclinical BP
Elboraey et al.^18^	A middle‐aged female	BNT162b1	Second dose	Stevens‐Johnson syndrome	New onset	5 days after vaccination	large, red‐colored lesions at the left retromolar area	**Retromolar area**: Large, deep‐red bullae **Labial mucosa**: Large oral ulcerations with yellow crust at the lower lip **Buccal mucosa**: Multiple large oral ulcerations	Oral prednisolone (30 mg/d) and oral corticosteroids in the form of a mouthwash (40 mg of triamcinolone acetonide + 100 mL of sterile saline)	T cell‐mediated immune reaction
Mansouri et al.^102^	A 49‐year‐old woman	BBIBP‐CorV	Second dose	Stevens‐Johnson syndrome	New onset	3 days after vaccination	Burning sensation in the mouth on the day of vaccine administration. The appearance of ulcers on her lips, and oral cavity three days after receiving the vaccine.	Ulcerations and erosions on the bilateral **buccal mucosa**, **lip mucosa**, **lower lip vermilion**, and over the **dorsal, lateral, and ventral surface of the tongue**.	Antihistamines (fexofenadine 180 mg daily) + Prednisolone (30 mg daily for a week then tapered off 10 mg every week)	T cell‐mediated immune reaction

### 5.1 Oral lichen planus

Oral lichen planus (OLP) is a chronic inflammatory condition that affects oral mucous membranes by inducing T‐cell‐mediated apoptosis of basal mucosal keratinocytes.^103^ COVID‐19 vaccines have been linked in recent publications to be attributable to either the new onset or worsening of OLP. Evidence suggests that T‐cell‐mediated immunity may be boosted by both mRNA and viral vector vaccinations.^104^ BNT162b2 vaccine has been shown to stimulate a strong CD8+ and T helper type 1 (Th1) cell response, as well as rising blood levels of IFN‐γ, TNF‐α, and IL‐2.^105^ Upregulation of Th1 cells and elevation of pre‐apoptotic cytokines, including IFN‐γ and TNF‐α have been reported as major agents responsible for the apoptosis of mucosal keratinocytes causing this autoimmune condition.^106^ It has been shown that the immune response to the Ad26.COV2.S vaccination remains for a longer time and has the potential to trigger autoimmunity.^107^

One study investigated potential risk factors for the development of oral lichen planus following immunization with mRNA‐based vaccines.^108^ Serologically positive patients receiving the first dose of BNT162b2 had 140 times higher IgG titers than their maximal pre‐vaccine levels. Moreover, seropositive persons have higher levels of antibody and T‐cell response potency.^109^ It has been suggested for those with a previous record of COVID‐19 infection that only a single dose of the BNT162b2 is recommended and would be sufficient to elicit a beneficial protective response with the lowest risk for incidence of adverse effects.^110^

### 5.2 Pemphigus vulgaris and bullous pemphigoid

Pemphigus vulgaris (PV) is a rare autoimmune disease that is characterized by painful blisters and erosions on the skin and mucous membranes. PV is thought to be a Th‐dominant disease with the contribution of CD8 + T cells via Fas/FasL signaling pathway.^111^ Two theories may explain how vaccination causes or triggers relapses of PV: The first suggests that some cases are genetically susceptible to hyper‐immunity. Another implies that vaccine antigens may cross‐react with antigens associated with PV pathogenesis.^112^ Receiving a second dose of mRNA‐based vaccines after the occurrence of autoimmune side effects following the first dose may be associated with the risk of worsening the condition, and administration of an alternative vaccine is highly recommended.^100^

Bullous pemphigoid (BP) is the most prevalent autoimmune subepidermal illness of the skin causing blisters, and it is sporadically accompanied by oral lesions.^113^ There have been cases reported with BP following the administration of SARS‐CoV‐2 mRNA‐based vaccines.^114^ Those who have an immunological predisposition or subclinical BP may be more susceptible to developing an autoimmune reaction after vaccination.^115^, ^116^

### 5.3 Stevens‐Johnson syndrome

Stevens‐Johnson syndrome (SJS) is an uncommon acute hypersensitivity immune reaction typically induced by medications causing severe necrosis of the skin and mucosal membranes. SJS is caused by a cytotoxic immunological response in keratinocytes that culminates in extensive keratinocyte apoptotic cell death.^117^ Although SJS is rarely associated with vaccinations, but second exposure to the causative agent leads to a recurrence that is typically more severe than the initial attack and may be fatal.^118^ Healthcare providers should be informed of this rare but severe condition to avoid receiving another dose of vaccine from the same type.

### 5.4 Oral manifestations of thrombocytopenia

Immune thrombocytopenia (ITP) is a type of autoimmune‐mediated reaction against platelets that can lead to a decrease in platelet count up to < 100 × 10^9^/L and subsequent submucosal bleeding with clinical manifestations of petechiae, purpura, excessive bruising, and bleeding.^119^ ITP can emerge secondary to COVID‐19 infection or even during postrecovery period, due to immune system dysregulation, and is managed by glucocorticoids and intravenous immunoglobulin treatment.^119^ Palatal petechiae have been reported in a case as an uncommon oral adverse effect of ChAdOx1 vaccine administration concomitant with a temporary reduction in platelet count to 140,000 platelets per microliter of blood which resolved 3 days later.^88^ Moreover, Lee et al. reported 20 patients with thrombocytopenia following BNT162b2 and mRNA‐1273 vaccination. Symptoms in the oral region were mostly petechiae, bruising, or gingival bleeding.^120^

## 6 DISCUSSION

COVID‐19 vaccination, though promising in pandemic control, have side effects. However, its positive outcomes outweigh potential side effects in large‐scale, and its administration can lead to defence against virus.

The majority of oral adverse reactions caused by anti‐SARS‐CoV‐2 vaccinations are self‐limited complications and resolve even without therapeutic interventions. In most of oral lesions and mucosal hypersensitivities, topical corticosteroids have proved to be effective in alleviating the patient's pain and accelerating the healing process. The emergence or exacerbation of autoimmune diseases with symptoms in the oral region after COVID‐19 vaccination but needs more caution. Cases of lichen planus or pemphigus vulgaris with oral lesions following COVID‐19 vaccination have been reported, for instance.

Immune response following viral infection has been largely studied as a triggering factor for the occurrence of autoimmune diseases.^121^ However, there is controversies regarding the same positive correlation to be found between various viral vaccinations and autoimmune responses. This notion is reported to be rare but possible, and susceptible patients with previous history of autoimmune diseases or having adverse reactions to other types of vaccines should be carefully assessed before viral vaccine administration.^122^

Vaccines against viral infections other than SARS‐CoV‐2 may also come with adverse effects with manifestations in the oral cavity. Similar to the anti‐SARS‐CoV‐2 vaccines, which have been widely administered after the outbreak, influenza vaccines are being received yearly, specifically by patients with systemic diseases.^123^ The results of a cohort study reported that there is no correlation between oral adverse effects and the administration of influenza vaccines.^124^ Nevertheless, some rare cases of autoimmunity have been reported for influenza vaccines. The cases appeared to be fewer than reported for anti‐SARS‐CoV‐2 vaccines.^112^ Lichen planus following hepatitis B vaccination might also be accompanied by oral mucosa involvement, specifically lips, buccal mucosa, or dorsal part of the tongue.^125^ HBsAg is assumed to have a causative role in this process due to the fact that surface epitopes on keratinocytes are similar or even identical to protein S of the hepatitis B virus as a vaccine component. This similarity consequently results in T‐cell activation and further immune responses to the mucosal cells.^126^ Lichenoid reaction is also reported in a few cases with the same involvement of protein S as an underlying mechanism.^127^, ^128^, ^129^ ITP is among other possible Hepatitis B vaccine‐related adverse reactions that mostly require no therapeutic interventions.^130^, ^131^ Measles, mumps, and rubella (MMR) vaccines are associated with a relatively low risk of acute ITP occurrence.^132^ Although the risk for ITP significantly increases in patients infected with each of the MMR viruses.^133^ Oral side effects following all viral vaccination should be considered carefully. Understanding these side effects is vital for the millions undergoing vaccination globally, as it not only influences clinical practices in managing postvaccination complications but also informs ongoing public health strategies.

Our research was critical for several reasons. It explored the scarcely examined territory of oral complications following COVID‐19 vaccination. Although these adverse effects are rare, they are of paramount importance given the vast numbers of vaccinated individuals globally. Autoimmune reactions and reactivations postvaccination is a critical area given the emerging reports following vaccine administration. By investigating these under‐researched facets, our study not only aims to bridge crucial knowledge gaps but also to provide pivotal insights for dentists and experts in this area.

Some limitations should be considered for this study. First and foremost, the studies on oral side effects following COVID‐19 vaccinations are mainly limited to case studies with few reported cases. There is a need for studies in large‐scale setting with higher levels of evidence. Given the novelty of research in this field, the correct frequency of each side effect remains unclear. Also, this study was a narrative review and does not follow a systematic approach. Therefore, the risk of selection bias should be considered.

## 7 CONCLUSION

COVID‐19 vaccination, though promising for infection prevention, are not without side effects. However, the advantages outweigh the disadvantages,

From a positive view, the vaccination can induce mucosal immunity. However, it is unclear whether this level of antibody produced by mucosa is protective. As IgA detected in saliva is primarily a product of mucosal immune response, more potent vaccines must be used to stimulate mucosal immunity to bring it up to protective levels. It is worth bearing in mind that mRNA‐based vaccines that induce a more robust mucosal immune response and SIgA secretion are associated with a higher risk of mucosal hypersensitivity and autoimmunity. Additionally, injectable vaccines raise concerns regarding the long‐term durability and efficiency of the mucosal immune response following vaccination, which is essential for inhibiting viral entrance through respiratory tracts. Several oral/nasal‐delivered mucosal vaccines have been proven to be effective in preventing respiratory infectious diseases such as COVID‐19. More research is needed to determine the effectiveness and possible side effects of these vaccines in human studies.

From a negative view, vaccination may cause oral lesions and inflammation as well as temporarily worsening pre‐existing disorders. Nevertheless, these side effects are manageable, and healthcare providers should not discourage patients from receiving the first vaccine in access. To the best of our knowledge, there is no established guideline for scheduling or discontinuing COVID‐19 vaccination for vulnerable cases. Therefore, the administration of another effective vaccine could be an appropriate decision, though we recommend further studies in this field to reach a consensus.

## AUTHOR CONTRIBUTIONS

**Shaghayegh Najary**: Conceptualization; writing–original draft. **Mohammadreza Vatankhah**: Methodology; writing–original draft. **Gita Khadivi**: Methodology; writing–original draft. **Seyyede N. Salehi**: Methodology; writing–original draft. **Mohammad A. K. Tabari**: Conceptualization; visualization; writing–review & editing. **Noosha Samieefar**: Conceptualization; supervision; writing–original draft; writing–review & editing. **Mohammad Behnaz**: Conceptualization; supervision; writing–review & editing.

## CONFLICT OF INTEREST STATEMENT

The authors declare no conflict of interest.

## TRANSPARENCY STATEMENT

The lead author Mohammad Behnaz affirms that this manuscript is an honest, accurate, and transparent account of the study being reported; that no important aspects of the study have been omitted; and that any discrepancies from the study as planned (and, if relevant, registered) have been explained.

## Data Availability

The data sets used and/or analyzed during the current study are available from the corresponding author on reasonable request.
